# Urinary Metabolomic Study in a Healthy Children Population and Metabolic Biomarker Discovery of Attention-Deficit/Hyperactivity Disorder (ADHD)

**DOI:** 10.3389/fpsyt.2022.819498

**Published:** 2022-05-20

**Authors:** Xiaoyi Tian, Xiaoyan Liu, Yan Wang, Ying Liu, Jie Ma, Haidan Sun, Jing Li, Xiaoyue Tang, Zhengguang Guo, Wei Sun, Jishui Zhang, Wenqi Song

**Affiliations:** ^1^Department of Clinical Laboratory, Beijing Children’s Hospital, Capital Medical University, National Center for Children’s Health, Beijing, China; ^2^Beijing Advanced Innovation Center for Big Data-Based Precision Medicine, Beihang University & Capital Medical University, Beijing, China; ^3^Proteomics Research Center, Institute of Basic Medical Sciences, School of Basic Medicine, Peking Union Medical College, Chinese Academy of Medical Sciences (CAMS), Beijing, China; ^4^Department of Mental Health, Beijing Children’s Hospital, Capital Medical University, National Center for Children’s Health, Beijing, China

**Keywords:** urinary metabolomics, childhood ADHD, biomarkers, healthy children, mass spectrometry

## Abstract

**Objectives:**

Knowledge of the urinary metabolomic profiles of healthy children and adolescents plays a promising role in the field of pediatrics. Metabolomics has also been used to diagnose disease, discover novel biomarkers, and elucidate pathophysiological pathways. Attention-deficit/hyperactivity disorder (ADHD) is one of the most common psychiatric disorders in childhood. However, large-sample urinary metabolomic studies in children with ADHD are relatively rare. In this study, we aimed to identify specific biomarkers for ADHD diagnosis in children and adolescents by urinary metabolomic profiling.

**Methods:**

We explored the urine metabolome in 363 healthy children aged 1–18 years and 76 patients with ADHD using high-resolution mass spectrometry.

**Results:**

Metabolic pathways, such as arachidonic acid metabolism, steroid hormone biosynthesis, and catecholamine biosynthesis, were found to be related to sex and age in healthy children. The urinary metabolites displaying the largest differences between patients with ADHD and healthy controls belonged to the tyrosine, leucine, and fatty acid metabolic pathways. A metabolite panel consisting of FAPy-adenine, 3-methylazelaic acid, and phenylacetylglutamine was discovered to have good predictive ability for ADHD, with a receiver operating characteristic area under the curve (ROC–AUC) of 0.918. A panel of FAPy-adenine, N-acetylaspartylglutamic acid, dopamine 4-sulfate, aminocaproic acid, and asparaginyl-leucine was used to establish a robust model for ADHD comorbid tic disorders and controls with an AUC of 0.918.

## Introduction

Metabolomics, an innovative analytical profiling technique, aims to detect the whole set of metabolites of low molecular weight present in body fluid. In recent years, it has already shown great potential in exploring the physiological status of healthy populations and in discovering subtle metabolic discrepancies in some specific disorders (1–4).

Urine samples can be collected through non-invasive protocols. Metabolic phenotyping of urine in a healthy population reflects the real-time dynamics of growth and development of the body and documents the physiological status of individuals (1, 2, 5). Some previous urinary metabolomic studies have revealed dynamic metabolic changes associated with age, sex, body mass index (BMI), dietary intake, and demographics in healthy children (1, 6–8), even in healthy neonates (2). Researchers have found that sex deeply influences both quantitative and qualitative urinary organic acid levels in healthy children aged 1–36 months, and the effect of sex is age-dependent (5). Metabolites that correlated with age included creatinine, creatine, glycine, betaine/TMAO, citrate, succinate, and acetone in children aged 12 years and younger (9). The metabolite profile of human urine also allows the prediction of sex and age with high accuracy in adults (10). Sili Fan et al. found that urinary metabolic signatures were globally distinct between healthy male and female children, and the levels of α-ketoglutarate and 4-hydroxybutyric acid increased 2.3-fold and 4.41-fold in male children compared to female children, respectively (11). In the urine of adult female children, succinate, citrate, hippurate, glycine, and malic acid are higher (11, 12), whereas creatine, stearate, alpha-ketoglutarate, and 4-hydroxy-butyrate are higher in healthy male children than in female children (11). Due to rapid growth and development in early life, the characteristics of the urinary metabolome are different between children and adults. However, large-scale metabolome studies in healthy children aged 1–18 years are lacking. A high-quality urinary metabolome for children of all ages is in great demand for investigating the metabolic changes in healthy children at each stage throughout early childhood, characterizing early-life physical and environmental exposures and assessing their general health status. Characterizing healthy children’s urinary metabolic features and their associations with age and sex can provide a standard reference metabolome, thus helping to assess disease metabolic disturbances and seek detectable biomarkers that differentiate health from disease.

Metabolomics has also been widely employed in identifying specific metabolic fingerprints in neuropsychiatric disorders in children, such as autism spectrum disorder (ASD) (13, 14) and attention-deficit/hyperactivity disorder (ADHD) (15). ADHD is a childhood-onset neurodevelopmental disorder marked by persistent and impaired inattention, hyperactivity/impulsivity, or both (16). It is one of the most common psychiatric disorders in childhood and adolescence, affecting approximately 7% of children worldwide (17), and it is more common in male children. ADHD adversely affects children’s emotional, behavioral, cognitive, academic, and social functions (18). Currently, the diagnosis of ADHD mainly depends on behavioral analysis, which is subjective and inconsistent, especially for children. It is imperative to investigate objective laboratory biomarkers for ADHD diagnosis. Recently, a few studies have proposed potential serum biomarkers, such as mono- and polyunsaturated fatty acids and the kynurenine pathway, in children and adults with ADHD, suggesting a potential linkage between metabolic characteristics and ADHD disease pathophysiology (15, 19–21). Bonvicini et al. performed a systematic review and meta-analysis of 6 biochemical studies and found lower serum docosahexaenoic acid (DHA) levels in adults with ADHD (19). Evangelisti et al. examined serum levels of tryptophan and other metabolites of the kynurenine pathway in children with ADHD. They found increased serum levels of tryptophan and kynurenine and reduced levels of kynurenic acid, anthranilic acid, and xanthurenic acid. The AUC of anthranilic acid was 0.88 (95% CI = 0.83–0.94) (22). In contrast to obtaining blood samples, urine is easy to collect in children due to its non-invasive procedures of collection. Urine can potentially provide crucial metabolic information. The applications of urine metabolomics are very promising in biomarker discovery for disease etiology, diagnosis, and prognosis (4, 23). However, there have been fewer urinary metabolomic studies on childhood ADHD.

In this study, we collected urine samples from 363 healthy children aged 1–18 years and 76 patients with ADHD (with or without comorbid tic disorders) for non-targeted metabolite profiling. We sought to (a) define metabolite associations with demographic factors, age, and sex in healthy children in a larger sample, (b) evaluate different urine metabolic profiles between patients with ADHD and healthy controls, and (c) compare different urine metabolic patterns between ADHD patients with/without tic disorders. We found that sex and age can influence interindividual variations in the urine metabolome of healthy children. Differences in urine metabolites were found in patients with ADHD. We constructed a differential metabolite pattern that could simultaneously discriminate three different types of psychiatric status (normal, ADHD, and ADHD with tic disorders). Furthermore, we discovered a biomarker panel that could distinguish ADHD from healthy controls with higher diagnostic values. Here, to the best of our knowledge, for the first time, we have identified novel urinary metabolites that could help to classify children with or without ADHD.

## Materials and Methods

### Cohort

All healthy participants aged 1–18 years were recruited through school voluntarily. Informed consent forms were obtained from each participant’s legally authorized representative (parent or guardian). The online health questionnaires based on the inclusion and exclusion criteria were completed by their guardians (24). Healthy volunteers were checked and examined by trained pediatricians according to standard operating procedures. All physical examination indices, including routine urine tests and biochemical tests, were in the normal range. Strict exclusion criteria, such as all types of genetic diseases and clinical laboratory values indicating acute or chronic disease, were applied. Based on physiological developmental characteristics of children and a previous study (24), we determined five age-specific partitions of the enrolled healthy participants for boys and girls: 1–3 years, 4–6 years, 7–10 years, 11–14 years, and 15–18 years of age.

A senior child psychiatrist interviewed the participants according to the Diagnostic and Statistical Manual of Mental Disorders, Fifth Edition (DSM-5), criteria (25). Conners’ parent rating scales were completed by each patient’s parents. A continuous performance test (CPT) (26) was administered to all the patients by a technician to obtain behavioral measures of attention. Patients with ADHD with comorbid tic disorders were examined by the Yale Global Tic Severity Scale (YGTSS) (27). Furthermore, experienced child psychiatrists conducted a neurocognitive assessment and physical examination to exclude any neurological disorder other than ADHD. Children with brain damage, a neurological disorder, a genetic disorder, epilepsy, or any other neurological disorder reported during the collection of personal history or anamnesis were excluded. Children who exhibited an IQ of 80 or lower according to the Combined Raven’s Test (CRT) or who were receiving drug treatment were also excluded. In this study, 76 outpatients with ADHD were recruited from Beijing Children’s Hospital. A total of 44 pediatric patients with ADHD and 32 children with both ADHD and chronic tic disorder were enrolled and randomly selected as the training group and validation group. The clinical features of the training and validation groups were similar. Biomarkers for ADHD with or without comorbid tic disorder were identified based on profiling analysis of 31 patients with ADHD, 21 ADHD patients with a tic disorder, and 46 age- and sex-matched healthy controls. An independent batch of patients with ADHD (13 ADHD and 11 ADHD with tic) and 17 healthy controls were used for external validation of the potential biomarkers using logistic regression.

### Urine Sample Collection and Preprocessing

Urine samples were collected and preprocessed according to guidelines (28, 29). Throughout the study, all urine samples were collected from the first urination in the morning under fasting conditions. In clean, dry specimen tubes from the same manufacturer, 10 ml of midstream samples were collected. Samples were centrifuged (3,000 g for 10 min) within 1 h of the collection; the supernatants were isolated, aliquoted, and stored at −80°C until analysis. Samples were shipped at cold temperatures. Freeze–thaw cycles were avoided.

### Urine Sample Preparation

Urine samples were prepared using the method described in our previous study (30). Briefly, 200 μl morning midstream urine samples were mixed with 400 μl acetonitrile to precipitate proteins. The mixture was vortexed for 30 s and centrifuged at 14,000 g for 10 min. After drying under vacuum, the supernatant was reconstituted with 200 μl 2% acetonitrile/water. Additionally, 10 kDa molecular weight cut-off ultracentrifugation filters (Millipore Amicon Ultra, MA, United States) were used to remove small proteins from the urine samples before transferring the samples to an autosampler.

The QC standard was prepared by mixing aliquots from all urine samples to assess the stability and repeatability of the analytical process.

### LC-HRMS Analysis

Urine sample analyses were conducted by a Waters ACQUITY H-class LC system coupled with an LTQ-Orbitrap mass spectrometer (MS) (Thermo Fisher Scientific, MA, United States). Urinary metabolites were separated with a 29-min gradient on a Waters HSS C18 column (3.0 mm × 100 mm, 1.7 μm) at a flow rate of 0.5 ml/min. Mobile phase A was 0.1% formic acid in H_2_O, and mobile phase B was acetonitrile. The gradient was set as follows: 0–1 min, 2% solvent B; 1–8 min, 2%–98% solvent B; 8–8.1 min, 98%–100% solvent B; 8.1–12 min, 100% solvent B; 12–12.1 min, 100%–2% solvent B; and 12.1–17 min, 2% solvent B. The column temperature was 45°C. The full MS acquisition ranged from 100 to 1,000 m/z at a resolution of 60 K in MS1 and 15 K in MS2. The MS1 automatic gain control target was 1 × 10^6^, and the maximum injection time (IT) was 100 ms. The MS2 automatic gain control target was set as 5 × 10^5^, and the maximum IT was 50 ms. The higher-energy collisional dissociation (HCD) fragmentation mode was used to dissociate differential metabolites with the optimal collision energy of 20, 40, 60, or 80 eV. Every urine sample was randomly injected into 3 technical replicates to reduce the experimental bias. All samples were randomly injected into the LC–MS system within a single analysis (within 12 days).

A quality control (QC) sample consisting of representative samples from populations with different genders and ages was used to monitor analytical performance throughout the run and was analyzed at an interval of every 10 samples. Overall, 42 injections were performed during the whole analysis. The analysis showed stable conditions with only a small variation (< ± 2 SD) (Supplementary Figure 1). These results provided some assurances that the platform had essential repeatability and stability throughout the analytical run.

### Data Processing

The raw data files obtained with LC-MS systems consist of a complex three-dimensional data format comprising retention time, m/z values, and density or abundance on each axis. This comprehensive information needs to be processed before any statistical techniques are applied to analyze the data. Data processing consists of certain steps, and one of them is converting the raw data produced by the instrument into a two-dimensional data matrix. Since data collection in metabolomic studies is carried out using special commercial software that works with LC-MS systems, the raw data obtained from the instruments should be converted to an open standard format such as mzML by using suitable software. After this conversion, the data can be used in many commercial and public programs to create data matrices using a public metabolomics software tool such as XCMS, Progenesis QI, and MetaboAnalyst. All these software are useful in terms of peak picking (EIC: extracted ion chromatography), deconvolution, and peak alignment. In this study, commercial software, Progenesis QI, was used for peak picking, alignment, and normalization. The data were normalized using “Normalize to the total compound.”

Further data preprocessing, including missing value estimation (50% rule), log transformation, and Pareto scaling, was carried out online using Metabo Analyst 5.0, a web-based tool^1^. Variables whose CV% (coefficient of variation) was more than 30% and missed in 50% or more samples were removed for further statistical analysis. Pattern recognition analysis (principal component analysis, PCA; orthogonal partial least squares discriminant analysis, OPLS-DA) was performed using SIMCA 14.0 (Umetrics, Sweden) software. The *Wilcoxon* rank-sum test was used to evaluate the significance of variables between the disease and control groups. Quantification of differentially expressed biomarkers was performed using EIC peak areas extracted and integrated by progenesis QI. Differential variables were selected according to the following rules: (1) adjusted *P*-value < 0.05, (2) fold change > 1.5, and (3) VIP value > 1.0.

### Feature Annotation and Metabolite Identification

Differential features were divided into several targeted lists. The lists were imported to the “MS2 method”, such as lists for targeted data-dependent analysis. The MS/MS spectra were further imported into Progenesis QI for metabolite annotation. In this study, two databases were used for MS/MS matching: (1) Metlin MS/MS library (Waters, version 1.0.6499.51447, commercial, composed of authentic standard spectra obtained by orbitrap and TOF mass spectrometry) and (2) fragment library constructed using theoretical fragments calculated by the theoretical fragmentation algorithm, the “MetFrag” algorithm (31). The detailed compound identification information (.csv file) included compound ID, adducts, formula, score, MS/MS score, mass error (in ppm), isotope similarity, theoretical isotope distribution, web link, and m/z values. Confirmation of the differential compounds was performed by the parameter of score value, calculated from mass error, isotope similarity, and fragmentation similarity (fragmentation score). The score value ranged from 0 to 60. According to the score results of the reference standards, the threshold was set at 35.0. Isotope similarity is calculated by comparing the measured isotope distribution of a precursor ion with the theoretical. The more reliable the compound identification, the higher the values obtained. Metabolite function annotation was performed using the KEGG metabolism pathway database, combined with manual annotation by reference searching. The predictive accuracy of biomarkers was assessed using the receiver operating characteristic (ROC) curve plotted in MetaAnalyst 4.0.

## Results

### Population Characteristics

**FIGURE 1:**
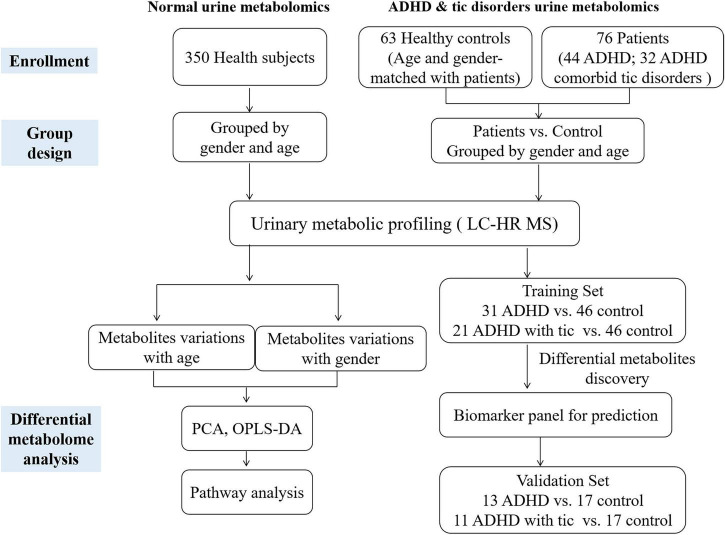
The workflow of this study.

We recruited a total of 350 healthy children (178 males and 172 females) aged 1–18 years, including five age stages (1–3 years, 4–6 years, 7–10 years, 11–14 years, and 15–18 years), 44 patients with ADHD, and 32 children with both ADHD and tic disorder. The workflow of our study is shown in Figure 1. The characteristics of the population included in this analysis are summarized in Table 1 and Supplementary Table 1. We analyzed urinary metabolic profiling and explored the metabolite variations associated with age and sex.

**TABLE 1:** Basic characteristics of the subjects enrolled in this study.

(1a) Numbers of healthy controls in different age stages			

Age stage	Male	Female	Total
Aged 1-3 (years)	29	25	54
Aged 4-6 (years)	30	29	59
Aged 7-10 (years)	39	40	79
Aged 11-14 (years)	39	39	78
Aged 15-18 (years)	41	39	80
Total	178	172	350

**(1b) Means and standard deviations for age used in analysis of the three groups**			

	**ADHD without tic disorders**	**ADHD comorbid tic disorders**	**Healthy control**

Cases	44	32	63
Age (years)	7.9 ± 2.0	8.7 ± 1.8	7.8 ± 1.8
Gender (Male/Female)	38/6	28/4	58/5

### Gender Variations in Children in Different Age Groups

**FIGURE 2:**
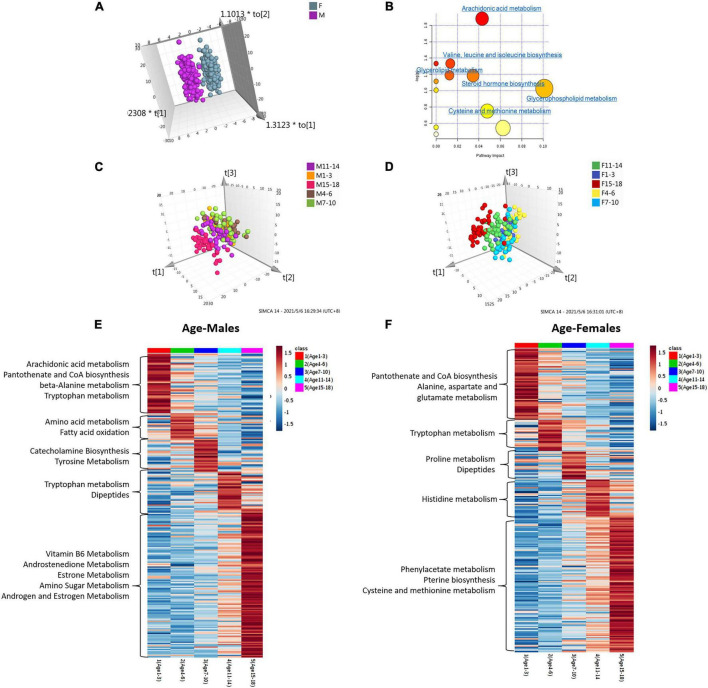
Analysis of metabolome interindividual variations and related factors (gender and age). **(A)** The score plot of the OPLS-DA model between female and male children of all ages. **(B)** Pathway overrepresentation analysis of differential metabolites in the two sex groups. The analysis was carried out with the metabolites in changes (*P* < 0.05). Pathway impact values were plotted against the *X*-axis, and *P*-values were plotted against the *Y*-axis. The node color is determined by its *P*-values, and the node size is proportional to the pathway impact values. (c,d) The score plot of the OPLS-DA model of male children **(C)** and female children **(D)** with different age stages. **(E,F)** Age-dependent metabolic pathways were enriched based on metabolites with the highest level in each age group. The KEGG database was the background pathway database.

We performed unsupervised principal component analysis (PCA) and supervised orthogonal partial least squares method-discriminant analysis (OPLS-DA) to explore the tendency of metabolic profiling variations between male and female children in five different age groups. The resulting score plot is shown in Supplementary Figure 2. Unsupervised PCA of different age groups showed the tendency that most male samples clustered together, although some male dots overlapped with the female samples (Supplementary Figure 2). Class separation could be obviously observed for all male and female children in five different age groups using an OPLS-DA model. Scatter plots showed that sex differences in urine metabolomics in each age group were apparent (Supplementary Figure 2 and Figure 2A). To validate the OPLS-DA model, the use of 100 permutation tests showed no overfitting of the models (Supplementary Figure 2).

Metabolites identified in sets with different age groups were selected by using the cut-off of OPLS-DA variable importance in projection (VIP) score of >1.0 and a *P*-value <0.05. The ratio of expression levels between female and male children is shown in Supplementary Tables 2A–F. We identified 73, 30, 28, 33, 69, and 64 metabolites to be significantly different between female and male children in sets aged 1–3, 4–6, 7–10, 11–14, and 15–18, and in all age groups, respectively (Supplementary Tables 2A**∼F**). Approximately half of the differential metabolites showed higher levels in female children, including 5′-methylthioadenosine, indoleacrylic acid, kynuramine, and tauroursodeoxycholic acid. Higher levels of the metabolites of L-Dopa, 3,4-methyleneazelaic acid, and 3-hydroxyhexanoyl carnitine, etc., were found in boys (Supplementary Tables 2A**∼F**).

We applied pathway enrichment analysis to analyze gender-dependent metabolism status in children. Figure 2B shows the sex-dependent metabolism pathways in all age groups. Arachidonic acid metabolism, valine-leucine-isoleucine biosynthesis, glycerolipid metabolism, etc., were found to be gender-dependent in children in different age groups (Figure 2B). Table 2 shows gender-dependent metabolism pathways of five different age groups: spermidine and spermine biosynthesis, estrone metabolism, etc., were found to be gender-dependent in healthy children aged 1–3 years; arachidonic acid and bile acid metabolism in children aged 4–6 years; glutamate, leucine, androsterone and fatty acid metabolism, etc., in children aged 7–10 years; proline and androsterone metabolism in children aged 11–14 years; and catecholamine, bile acid, estrone, and tyrosine metabolism, etc., in children aged 15–18 years.

**TABLE 2:** Gender dependent metabolism pathways of five age groups.

Age stages	Metabolism pathways
Aged 1-3 (years)	Spermidine and spermine biosynthesis; Estrone metabolism; Mitochondrial beta-oxidation of short chain saturated fatty acids; Propanoate metabolism; Methionine metabolism; Valine, leucine and isoleucine degradation
Aged 4-6 (years)	Arachidonic acid metabolism; Bile acid metabolism
Aged 7-10 (years)	Glutamate metabolism; Bile acid metabolism; Fatty acid metabolism; Leucine metabolism; Androsterone metabolism
Aged 11-14 (years)	Leukotriene E4 metabolism; Proline metabolism; Androsterone metabolism
Aged 15-18 (years)	Catecholamine biosynthesis; Estrone metabolism; Bile acid biosynthesis; Androgen and estrogen metabolism; Tyrosine metabolism

### Age Variations in Female and Male Groups

Both the OPLS-DA and PCA models showed the obvious tendency of metabolic profiling variations with age (Figures 2C**,D** and Supplementary Figure 3). Five age groups, including 1–3 years, 4–6 years, 7–10 years, 11–14 years, and 15–18 years, were compared. Both the female and male groups showed the same age-dependent metabolic status (Figures 2C,D). Notably, one hundred permutation tests showed no overfitting of the two models (Supplementary Figure 3).

According to the significance threshold, 250 metabolites were identified as key molecules with a significant correlation with age in the boy group (Supplementary Table 3) and 243 metabolites were identified in the girl group (Supplementary Table 4). These differential metabolites were submitted for further pathway analysis. Figures 2E,F show the relative intensity change trend among the five age groups of male and female children, respectively. Catecholamine biosynthesis, pantothenate and CoA biosynthesis, vitamin B6, androstenedione, and estrone metabolism were found to change with age in boys. In girls, metabolites involved in phenylacetate metabolism, pantothenate and CoA biosynthesis, and pterin biosynthesis were age-dependent (Table 3 and Figures 2E,F).

**TABLE 3:** Age dependent metabolism pathways in male and female children.

Gender	Age-dependent metabolism pathways
Male	Arachidonic acid metabolism, Pantothenate and CoA biosynthesis, Beta-alanine metabolism, Tryptophan metabolism, Amino acid metabolism, Fatty acid oxidation, Catecholamine biosynthesis, Tyrosine metabolism, Tryptophan metabolism, Dipeptides, Vitamin B6 metabolism, Androstenedione metabolism, Estrone metabolism, Amino sugar metabolism, Androgen and estrogen metabolism
Female	Pantothenate and CoA biosynthesis, Alanine, aspartate and glutamate metabolism, Tryptophan metabolism, Proline metabolism, Dipeptides, Histidine metabolism, Phenylacetate metabolism, Pterin biosynthesis, Cysteine and methionine metabolism

### Attention-Deficit/Hyperactivity Disorder Biomarker Discovery

From the above results, we found that age and sex are important factors affecting metabolism, so we used age- and sex-matched healthy control subjects to analyze urine metabolomics differences between the ADHD and control groups. Both unsupervised PCA and supervised OPLS-DA suggested apparent discrimination between the two groups (Supplementary Figures 4A**∼B**). Differential metabolites were selected according to the VIP value (VIP > 1). Notably, sixty significantly differential metabolites were identified (Supplementary Table 5). The data indicate that metabolites involved in dihydrolipoamide, 3-methylazelaic acid, and phenylacetylglutamine were upregulated in patients with ADHD, whereas the metabolites isohomovanillic acid, indanone, and dopamine 4-sulfate were downregulated.

ROC curves were used to evaluate the diagnostic accuracy of the differential metabolites for ADHD. A metabolite panel consisting of FAPy-adenine, N-acetylaspartylglutamic acid, and dopamine 4-sulphate was found to have the best prediction accuracy for ADHD. The area under the curve (AUC) was 0.923 for the training set (Table 4), the sensitivity was 94.2%, and the specificity was 82.6%. Furthermore, an independent batch of patients with ADHD (13 with ADHD and 11 ADHD with tic) and 17 healthy controls were used for external validation of the potential biomarkers. External validation achieved an AUC of 0.877 (Table 4 and Supplementary Figure 4C).

**TABLE 4:** ROC of three groups.

	Disease vs. Normal^a^	ADHD vs. Normal^b^	ADHD comorbid tic disorders vs. Normal^c^
	AUC	AUC	AUC
Training Set	0.923	0.918	0.918
Validation Set	0.877	0.96	0.918

*a The panel includes FAPy-adenine, N-Acetylaspartylglutamic acid and Dopamine 4-sulfate.*

*b The panel includes FAPy-adenine, 3-Methylazelaic acid and Phenylacetylglutamine.*

*c The panel includes FAPy-adenine, N-Acetylaspartylglutamic acid, Dopamine 4-sulfate, Aminocaproic acid and Asparaginyl-Leucine.*

**FIGURE 3:**
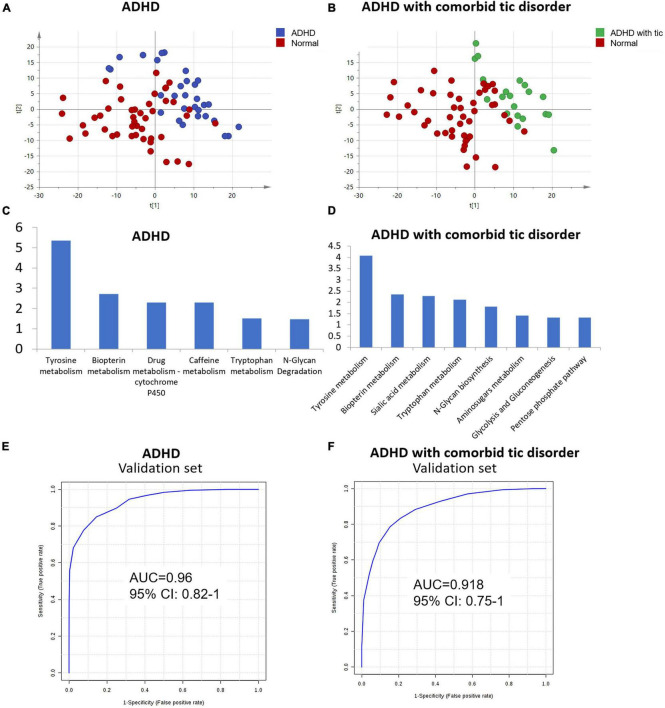
Analysis of metabolome interindividual variations and related factors. **(A)** The score plot of the PCA model between ADHD without tic disorder and normal controls. **(B)** The score plot of PCA based on urine profiling of ADHD comorbid with tic disorder and normal controls. **(C,D)** Enriched pathway of metabolites for ADHD with/without tic disorder. **(E,F)** ROC plot of the validation set of ADHD patients with/without tic disorder.

Furthermore, we explored the urine metabolic differences between ADHD without tic disorder and healthy controls. PCA was performed, and the analysis showed apparent discrimination between the control samples and the ADHD disease groups (Figure 3A). Furthermore, an OPLS-DA model established for differential metabolite selection also showed apparent discrimination (Supplementary Figure 5A). A total of 34 significantly differential metabolites were identified in children with ADHD without tic disorders (Supplementary Table 6). Pathway power analysis indicated that tyrosine metabolism, biopterin metabolism, drug metabolism-cytochrome P450, caffeine metabolism, tryptophan metabolism, and N-glycan degradation were differentially regulated in ADHD (Figure 3C). A metabolite panel consisting of FAPy-adenine, 3-methylazelaic acid, and phenylacetylglutamine was used to construct a robust model for distinguishing between the healthy group and the ADHD without tic disorder group. The AUC of the panel was 0.918 for the training set (Table 4), and its sensitivity and specificity were above 0.8 (Supplementary Figure 6A). The AUC for the external validation set was 0.96 (Table 4 and Figure 3E).

Using the same strategy as above, PCA and OPLS-DA models were performed to visualize the metabolomic differences between ADHD comorbid with tic disorder and healthy control subjects. Both PCA and OPLS-DA showed apparent discrimination between the two groups (Figure 3B, Supplementary Figure 5B). Then, 42 differential metabolites were identified in children with ADHD with comorbid tic disorders (Supplementary Table 7). These differential metabolites involve pathways of tyrosine metabolism, biopterin metabolism, sialic acid metabolism, tryptophan metabolism, N-glycan biosynthesis, amino sugar metabolism, glycolysis, gluconeogenesis, and pentose phosphate metabolism (Figure 3D). A metabolite panel consisting of FAPy-adenine, N-acetylaspartylglutamic acid, dopamine 4-sulfate, aminocaproic acid, and asparaginyl-leucine was found to have a good distinction between the healthy group and the ADHD with tic disorder group. The AUC-ROC of the panel was 0.918 for the training set (Table 4), and it achieved sufficient sensitivity (0.83) and higher specificity (0.91) (Supplementary Figure 6B). The AUC-ROC was 0.918 for the external validation set (Table 4 and Figure 3F).

In addition, we examined the main metabolic characteristics of ADHD patients with and without comorbid tic disorder and healthy controls. Metabolites showing the highest level for each group were submitted for pathway analysis, indicating the specific metabolic characteristics for each group. Arginine biosynthesis, pentose and glucuronate interconversions, and alanine, aspartate, and glutamate metabolism were the main metabolic features in the healthy group. Compared with the control group, the two disease groups showed similar metabolic features. Leucine metabolism and fatty acid metabolism showed higher activity in ADHD patients with a tic disorder. In ADHD without tic disorder, glutamate metabolism was active (Supplementary Figure 7).

## Discussion

The analytical platforms to be used for metabolomic studies should be able to simultaneously analyze hundreds of metabolites from complex biological samples and also allow monitoring of changes in these metabolites. However, none of the current analytical platforms available today has the power to fully measure the whole metabolome, and this may be due to the physicochemical diversity of these metabolites, e.g., hydrophilic carbohydrates, volatile alcohols and ketones, amino and non-amino organic acids, and hydrophobic lipids. Analytical techniques, such as NMR, GC-MS, and LC-MS, are the most commonly used analytical methods for metabolomic studies. Different analytical methods showed differences in resolution and sensitivity, which would contribute to the number of identified metabolites. In this study, the LC-MS method was used for biomarker discovery. LC-MS shows higher resolution and sensitivity than NMR and GC-MS and could identify more metabolites (32, 33).

The spectra data files obtained with LC-MS systems consist of a complex three-dimensional data format, comprising retention time, m/z values, and density or abundance on each axis. Data processing consists of certain steps, and one of them is converting the raw spectra data produced by the instrument into a two-dimensional data matrix, named peak picking and alignment procedures. Several tools, including XCMS, Progenesis QI, and MetaboAnalyst, could perform peak picking. The accuracy of peak picking is imperative in the analysis of LC-MS-based metabolomic data (34). In this study, peak alignment was carried out automatically, using a QC run as the reference. The alignment vector is used for quality control. The quality evaluation results indicated that the score values for all the samples were greater than 80% (35).

Compared to previous adult urine metabolomic studies, there are relatively few studies on healthy children. Due to rapid growth and development in early life, each age range in the childhood period has different metabolic features. In this study, we characterized the urine metabolic profiles of a large sample size of healthy children among five different age groups (from 1 to 18 years of age) using the LC–MS/MS platform. Our results showed that the urinary metabolic signature of children was associated with sex and age parameters. Urine is a sensitive matrix that can reflect physiological and pathological changes. Urine metabolomics reflects the disturbance in disease states and is commonly used for the diagnosis of neuropsychiatric diseases. Urine samples are routinely collected easily from young children. Our study identified seven urinary metabolites that could be used as effective biomarker panels for ADHD diagnosis and could help to elucidate the underlying molecular pathological mechanisms of the disease.

### Gender-Dependent Metabolism Status in the Healthy Children Population

Previous studies have highlighted the impact of sex on the urinary metabolome in adults (10, 36–38). Manuela J. Rist et al. found that urine metabolite profiles could predict sex in healthy adults with an accuracy of prediction of approximately 90% (10). Several studies also underlined that sex was one of the most relevant biological variables significantly influencing metabolomic profiles in children. Scalabre and coworkers (7) investigated the influence of age on the newborns’ urine metabolome during the first 4 months of life using ^1^H-NMR spectroscopy combined with multivariate statistical analyses. They did not find any statistically significant differences between male and female children. However, Caterino et al. (5) investigated sex influences on 72 organic acids measured through GC–MS analysis in the urine of 291 children aged 1–36 months and stratified them into four age groups. They demonstrated that sex deeply influenced urinary organic acid levels, and the sex-induced variations depended on age. López-Hernández and their team (2) identified and quantified 136 metabolites in the urine of 48 healthy neonates collected in the first 24 h of life, and sex differences were found for 15 metabolites.

In this study, we included healthy children aged 1–18 years and almost equal numbers of healthy male and female subjects (50.9% boys/49.1% girls). Interestingly, our results suggest that gender differences in the urinary metabolome are already present during childhood, even from the early years of life (1–3 years of age). Our pathway analysis revealed strong differences in steroid hormone biosynthesis and valine-leucine and isoleucine biosynthesis between female and male children in all age groups. In addition, our results showed that the influence of sex was linked to age, and the single age group presented some specificity. The metabolic phenotypes of boys showed the presence of significantly higher concentrations of 3-hydroxydodecanoyl carnitine and 3-hydroxyhexanoyl carnitine and lower 11-beta-hydroxyandrosterone-3-glucuronide and valyl-proline compared to girls in all age groups. Carnitine plays important role in fatty acid oxidation and branched-chain amino acid metabolism. It can facilitate fatty acids to shuttle the mitochondrial membrane by combining with fatty acids to form acyl-carnitines. 3-Hydroxydodecanoyl carnitine and 3-hydroxyhexanoyl carnitine belong to the family of acyl-carnitines, which are beta-oxidation products of fatty acids (39). Previous studies have reported that healthy adult male subjects (19–69 years) had higher levels of urinary metabolites related to fatty acid oxidation (carnitine, acetylcarnitine) than female subjects (38), and newborns had a markedly increased acylation degree of carnitine in urine compared with healthy boys (8–15 years) (40). In this study, we confirmed gender-specific acylcarnitine metabolites in urine in younger children aged 1–3 years. Elevated carnitine and its related metabolites in male children relative to female children may suggest a greater usage of fats for energy metabolism for male children at rest vs. female children (38).

### Age-Dependent Metabolism Status in the Healthy Children Population

Urine and serum metabolomics were reported to reveal dynamic metabolic changes associated with age both in children (1, 7–9) and in the adult population (10, 36, 41). However, to date, most metabolome studies in children have covered a relatively narrow age range. Here, we provided an overview of the dynamic metabolic changes in a large cohort of healthy children, comprising all age groups from 1 to 18 years of age, which potentially provided complete information on rapid physical growth during the childhood period. Since we observed that sex played an important role in urine metabolism profiling, we analyzed age variation in boys (males) and girls (females). We found that pantothenic acid urinary concentration decreases with age both in boys and girls. Pantothenic acid is an essential micronutrient and serves as a cofactor in the synthesis of coenzyme A, which is essential for the metabolism and synthesis of the TCA cycle and fatty acid oxidation (42). Aureìlien Scalabre et al. also observed that urinary pantothenate decreased with age and weight in newborns under 4 months of age (7).

In addition, we found that the pathways of catecholamine biosynthesis, vitamin B6, estrone, and androstenedione metabolism changed with age in boys; phenylacetate metabolism and pterin biosynthesis were age-dependent in girls. Age-related differences in urinary catecholamine excretion both in adults and in children have been reported by several studies (43–47). There was a sex difference, with lower values in girls and women than in their male counterparts (48, 49). The 24-h urinary excretion of dopamine (DA) was significantly inversely related to age in adult women but not in men (47). However, Anne et al. found that there was a linear relationship between age and the excretion of the urinary catecholamine metabolites epinephrine (E), DA, vanillylmandelic acid (VMA), and homovanillic acid (HVA) in children aged 3–16 years (45). Dalmaz et al. also determined catecholamines (DA, E, and norepinephrine (NE)) in human urine from one day of age to adulthood. They observed that the maturation process of the sympathoadrenal system is not achieved at birth; both sympathetic and glandular function remain low during a prolonged period in childhood, reaching full maturation probably near the fifth year of life (44). Here, we found that urinary DA, HVA, and 6-hydroxydopamine levels changed with age in males/boys, reaching a peak at the age of 7–10 years. In addition, androstenedione, androstanol, and their glucuronide metabolites showed high levels during prepubescence (11–14 years) and puberty (15–18 years). Our results demonstrated that age-dependent metabolic profile changes revealed not only rapid growth occurring in childhood but also sex maturation.

### Attention-Deficit/Hyperactivity Disorder Biomarker Discovery

Currently, metabolic strategies have been used to characterize specific metabolic phenotypes associated with ADHD disorders, and several metabolites have been identified (3, 15, 19). The urinary metabolism of ADHD has not been sufficiently investigated thus far in children. It is essential to determine an accurate and sensitive non-invasive diagnostic biomarker of ADHD for children in whom disease diagnosis is more difficult. In our study, a panel based on three urinary metabolites was found to have high accuracy for ADHD disease, and a pattern based on three urinary metabolites, namely, FAPy-adenine, 3-methylazelaic acid, and phenylacetylglutamine, was found to have high sensitivity and specificity for ADHD without tic disorders, as was a panel based on five urinary metabolites for ADHD with tic disorders.

We compared the urinary metabolic profile between the pediatric ADHD cases and the age- and gender-matched healthy controls, and we observed a total of 60 metabolites that could significantly differentiate cases with ADHD from the healthy controls. Our metabolic pathway analysis showed that amino acid metabolism (e.g., L-norleucine and citrulline) and fatty acid metabolism (e.g., aminocaproic acid and 3-methylazelaic acid) pathways were associated with ADHD. Previous ADHD studies have suggested that metabolites in blood serum involved in fatty acids contribute to the distinction between adults diagnosed with ADHD and control groups (21, 50). In our study, we found that the content of 3,4-methylenepimelic acid in urine was decreased in ADHD groups, and aminocaproic acid and 3-methylazelaic acid were increased in patients with ADHD. Although the specific pathological mechanisms of these fatty acids are not yet clear, our results indicated that fatty acid metabolism in patients with ADHD was disturbed.

Dopamine (DA) is a member of the catecholamine family of neurotransmitters. The correlation between DA and ADHD is the most widely studied. DA is synthesized by converting tyrosine into levodopa (L-DOPA), which can then be converted into DA. Extensive neurobiological, pharmacological, and neuroimaging evidence suggests that ADHD is characterized by defects in DA production or metabolic disorders (44, 45). In our study, we found that urinary metabolites belonging to the tyrosine metabolic pathways displayed the largest differences between patients with ADHD and healthy controls. The change in urinary metabolites involving tyrosine metabolic pathways may indirectly reflect dopamine metabolic profile alterations in ADHD. Moreover, we found that the urinary levels of the main degradation metabolites of DA, dopamine 4-sulfate and isohomovanillic acid, were decreased in children diagnosed with ADHD. Sulfation is one of the major degradative pathways of DA, and the main excretion products of DA found in human urine are homovanillic acid and its sulfates (46). Several studies have found changes in urinary levels of dopamine and its metabolites (homovanillic acid, dihydroxyphenylalanine, and dihydroxyphenylacetic acid) in patients with ADHD (47). Our results are consistent with previous reports and indicate that urinary DA metabolites could be potential diagnostic candidates. However, taking into consideration the complexities of dopamine production and metabolism, the relationship between urinary metabolic alternation and the molecular mechanism of ADHD remains to be fully elucidated. Urinary nucleosides and deoxynucleosides are mainly known as metabolites of RNA turnover and oxidative damage of DNA. FAPy-adenine is an oxidized DNA base. Oxidized nucleosides are biochemical markers for some neurodegenerative diseases (Alzheimer’s disease) (51). Combined with the other two metabolites, dopamine 4-sulphate and N-acetylaspartylglutamic acid, FAPy-adenine obtained higher diagnostic accuracy (ROC-AUC was above 0.8). However, its pathological mechanism in ADHD needs to be explored further.

## Conclusion

This study provided an overview of the dynamic urinary metabolic changes in children from 1 to 18 years of age. These results may be potentially useful in assessing the biological age (as opposed to chronological) of young humans as well as in providing a deeper understanding of the confounding factors in the application of metabolomics. Such a large-scale cohort may pave the way for the future building of urine metabolomic reference profiles in healthy children and may provide insight into the complex metabolic changes in children’s growth and development.

ADHD is more difficult to diagnose in children, as the disease diagnosis depends on the parent’s scale and physicians’ diagnostic procedure, which has been criticized for strong subjectivity. Here, our pilot study encouraged the application of a panel of metabolites in ADHD diagnosis and offered the opportunity to standardize and improve disease diagnostic assessment.

Our study has several limitations that should be mentioned. First, while the ADHD-specific urinary metabolites were validated in one independent sample cohort in our study, the sample size was still relatively small, which may have reduced the statistical power of the study. Our findings need to be further validated with a larger sample. In the future, we will collect more samples to validate the findings. Second, we found urinary metabolite markers between children with ADHD and healthy controls; however, the causal relationships between these peripheral biomarkers and central nervous system disease remain unknown. Furthermore, we will carry out the functional study of these urinary metabolites in animal models of childhood ADHD. It could increase our understanding of the pathological mechanism of ADHD.

## Data Availability Statement

The original contributions presented in the study are included in the article/Supplementary Material, further inquiries can be directed to the corresponding authors.

## Ethics Statement

The studies involving human participants were reviewed and approved by the Ethics Committee of Beijing Children’s Hospital. Written informed consent to participate in this study was provided by the participants’ legal guardian/next of kin. Written informed consent was obtained from the individual(s), and minor(s)’ legal guardian/next of kin, for the publication of any potentially identifiable images or data included in this article.

## Author Contributions

XTi, XL, JL, and XTa performed the experiments. ZG, HS, and YL verified the methodology. YW, YL, and JM collected urine samples and clinical data. XTi and XL wrote the manuscript. WSu, JZ, and WSo designed the study. All authors contributed to the article and approved the submitted version.

## Conflict of Interest

The authors declare that the research was conducted in the absence of any commercial or financial relationships that could be construed as a potential conflict of interest.

## Publisher’s Note

All claims expressed in this article are solely those of the authors and do not necessarily represent those of their affiliated organizations, or those of the publisher, the editors and the reviewers. Any product that may be evaluated in this article, or claim that may be made by its manufacturer, is not guaranteed or endorsed by the publisher.

## Footnotes

1
http://www.metaboanalyst.ca
