# Supplementary figures and images for: Urinary Metabolomic Study in a Healthy Children Population and Metabolic Biomarker Discovery of Attention-Deficit/Hyperactivity Disorder (ADHD)
Source: Front Psychiatry. 2022 May 20;13:819498. doi: 10.3389/fpsyt.2022.819498 (PMC9163378; doi:10.3389/fpsyt.2022.819498)

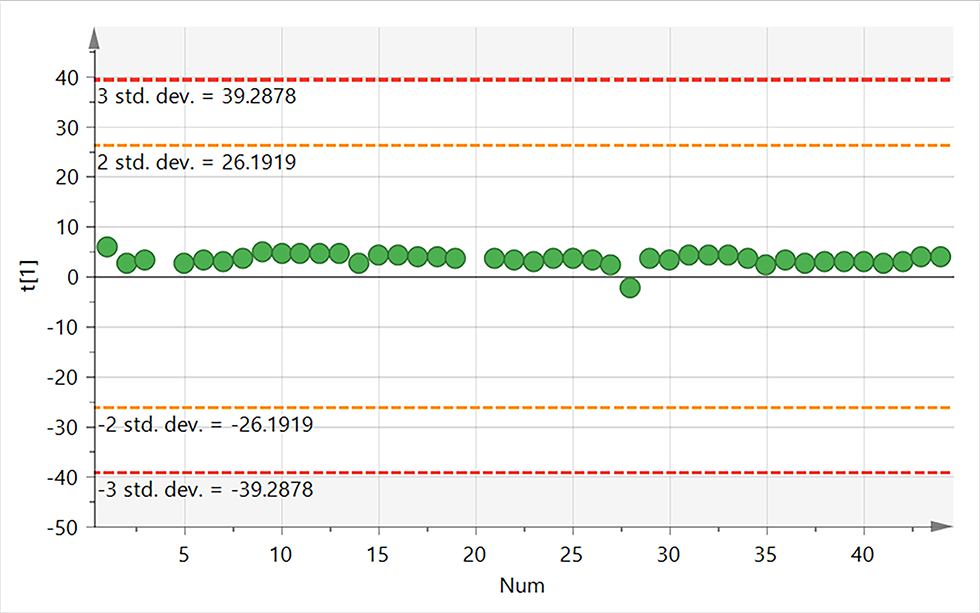

Supplement: Supplementary file 1 [file Image_1.TIF]

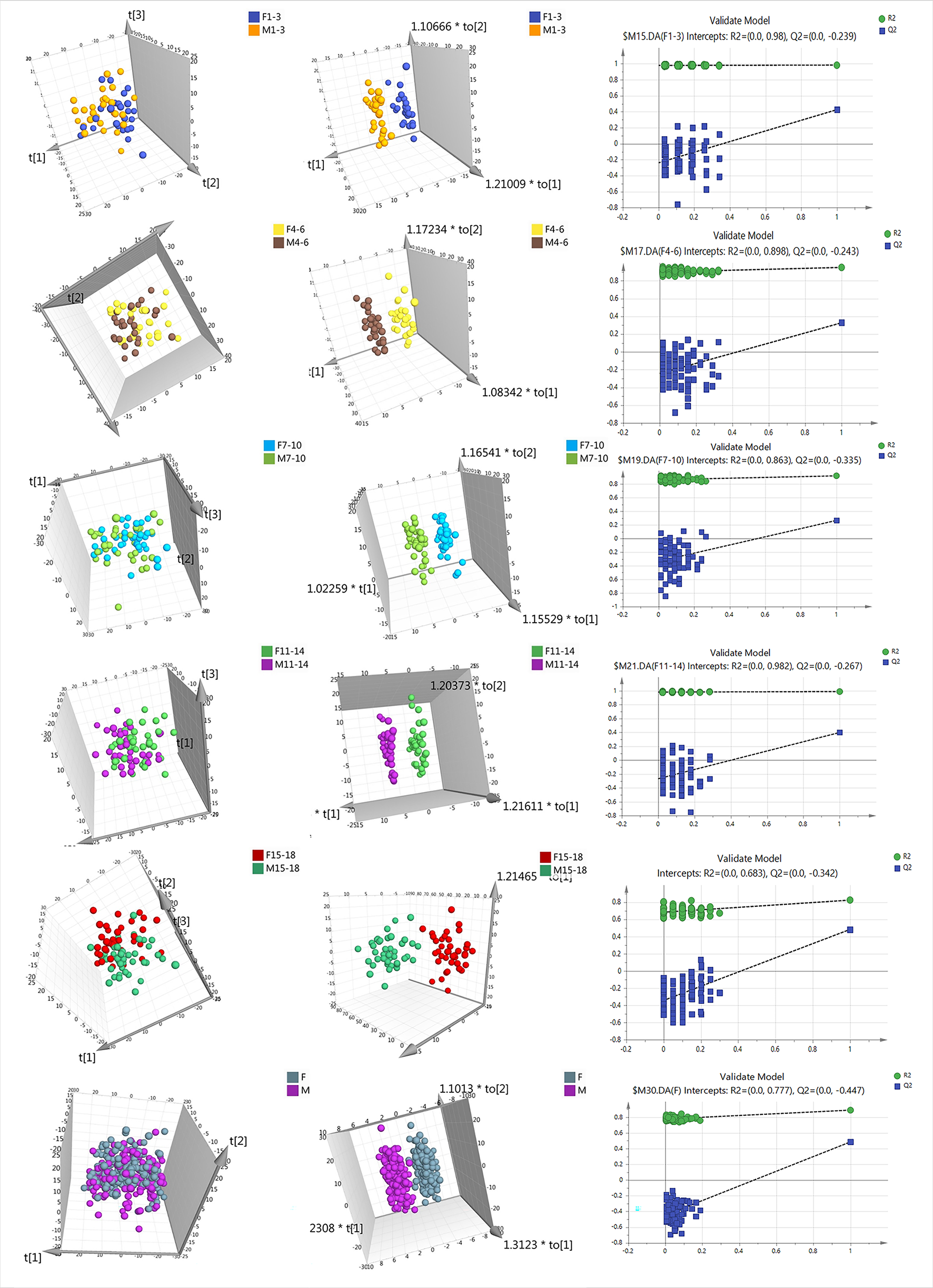

Supplement: Supplementary file 2 [file Image_2.TIF]

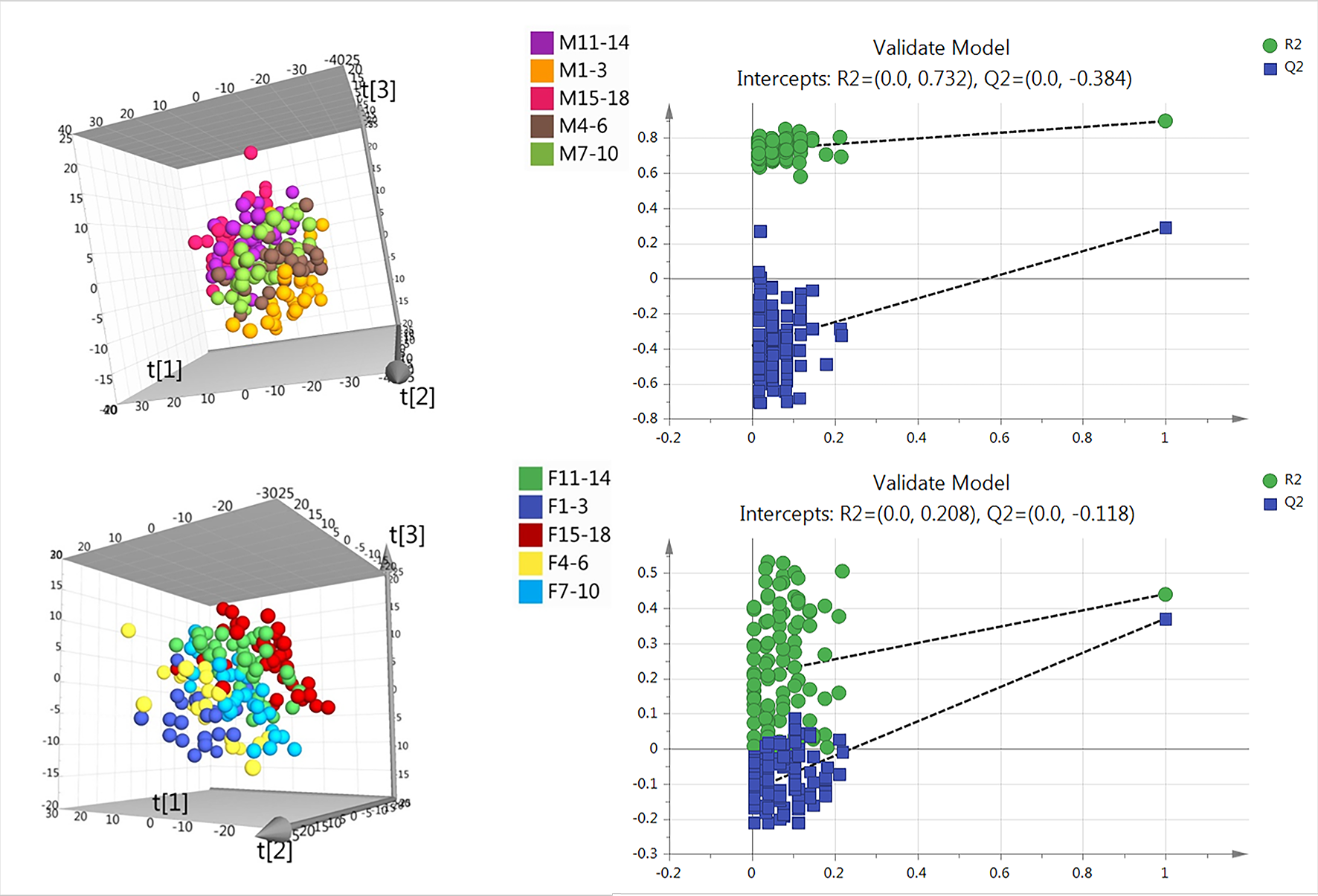

Supplement: Supplementary file 3 [file Image_3.TIF]

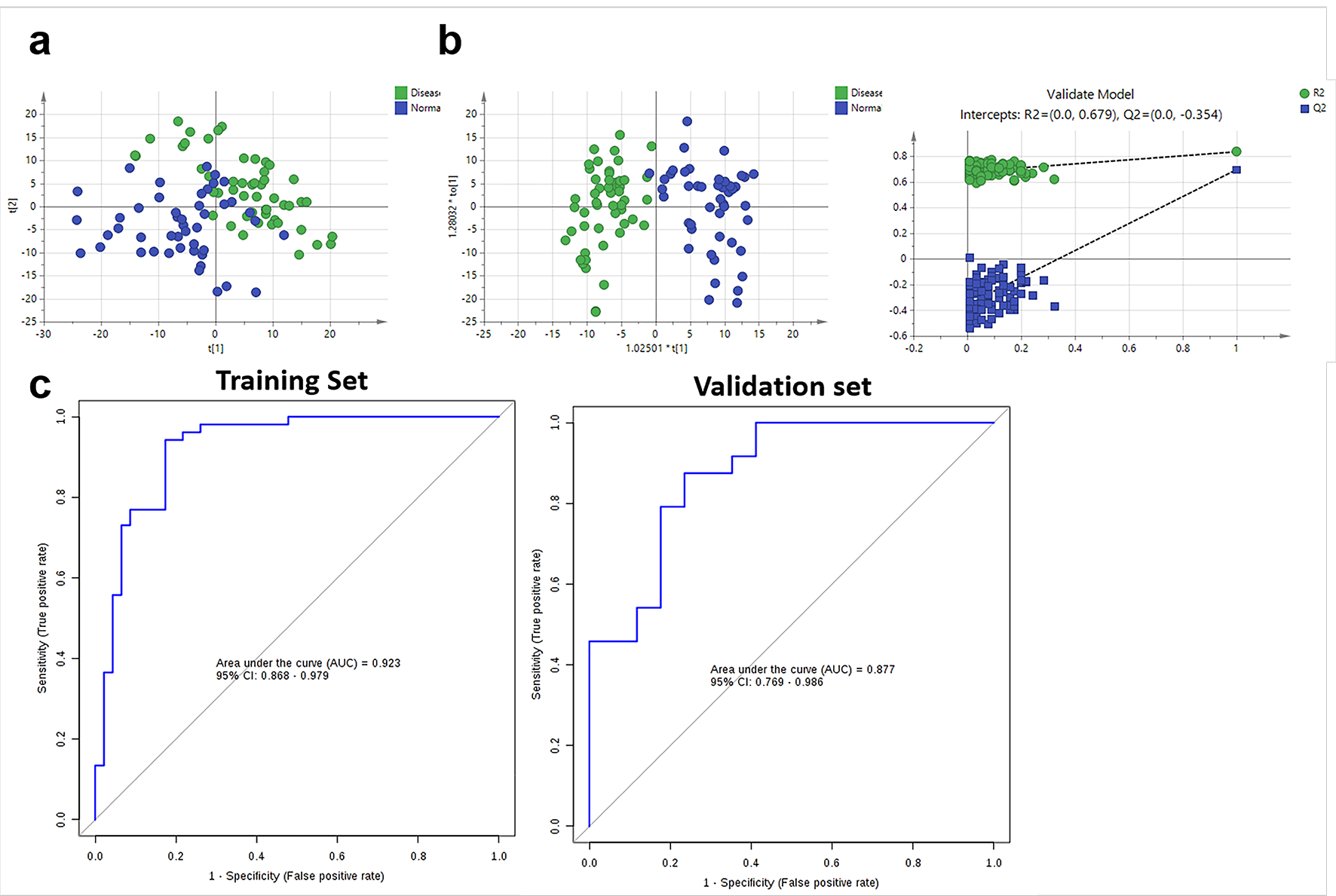

Supplement: Supplementary file 4 [file Image_4.TIF]

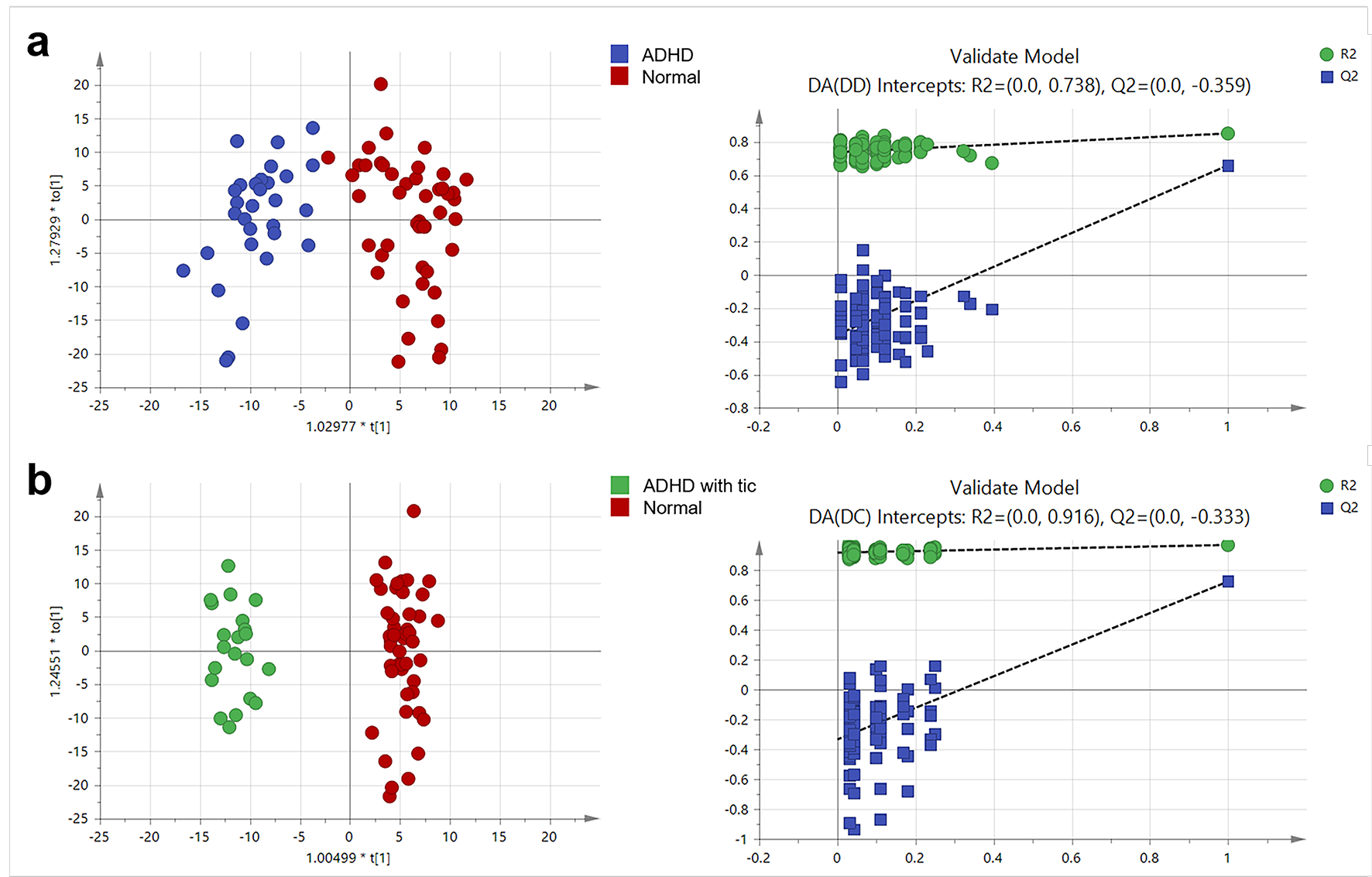

Supplement: Supplementary file 5 [file Image_5.TIF]

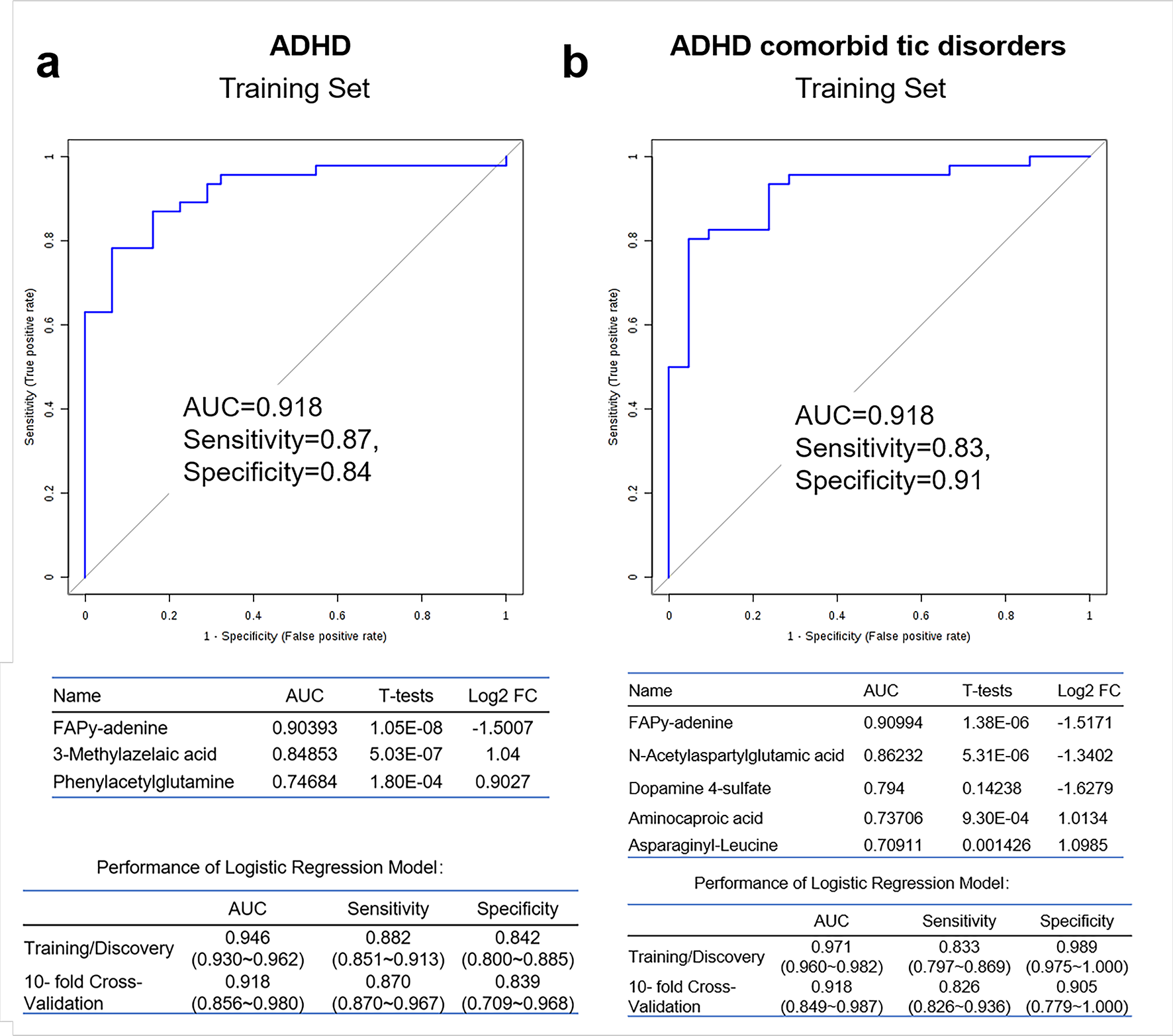

Supplement: Supplementary file 6 [file Image_6.TIF]

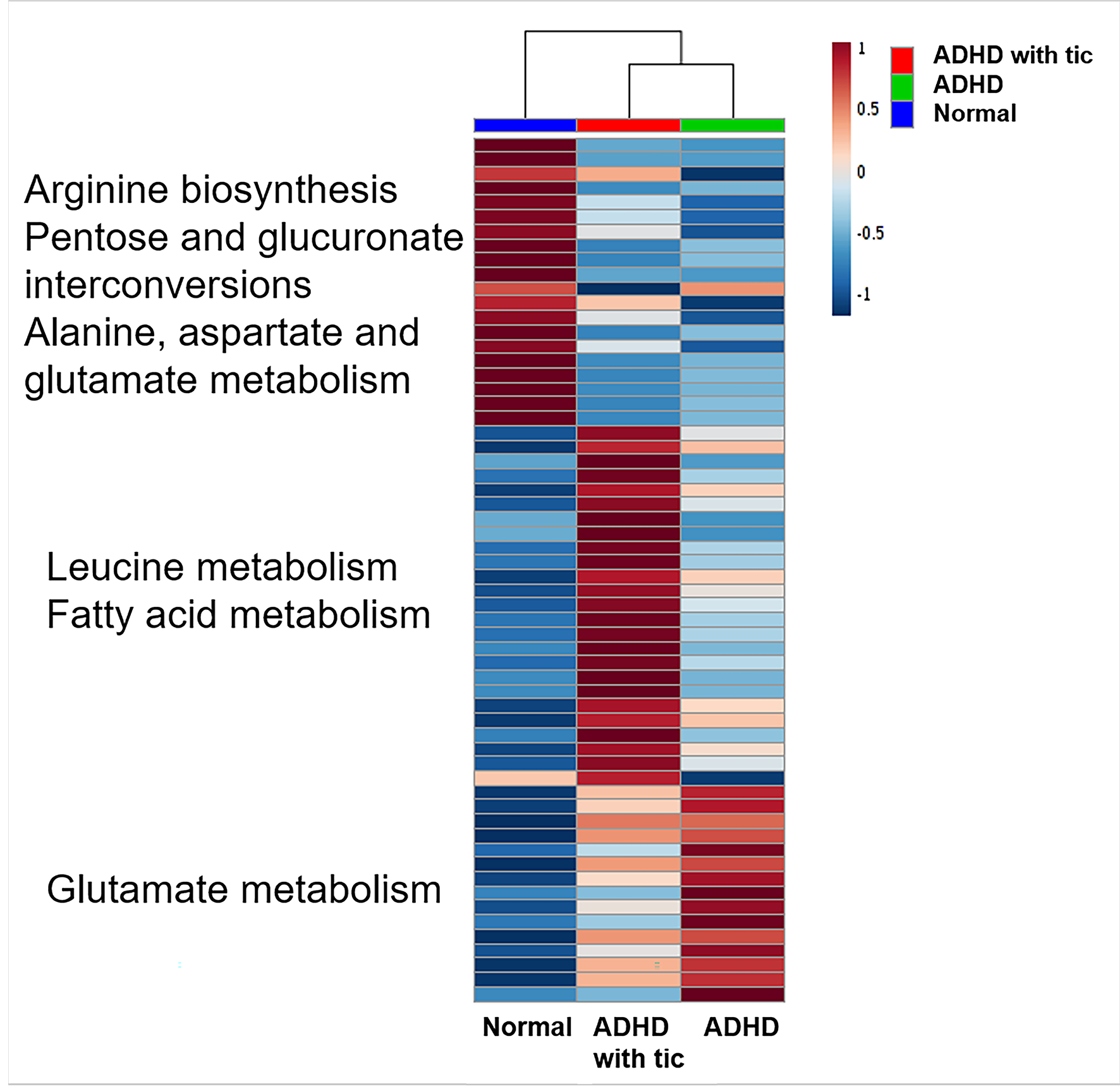

Supplement: Supplementary file 7 [file Image_7.TIF]
