# Supplementary material for: Urinary Metabolomic Study in a Healthy Children Population and Metabolic Biomarker Discovery of Attention-Deficit/Hyperactivity Disorder (ADHD)
Source: Front Psychiatry. 2022 May 20;13:819498. doi: 10.3389/fpsyt.2022.819498 (PMC9163378; doi:10.3389/fpsyt.2022.819498)
Supplement: Supplementary file 9 [file Data_Sheet_2.docx]

**Investigation of children urinary metabolomic characteristics and metabolic biomarker discovery of attention deficit/hyperactivity disorder (ADHD)**

Xiaoyi Tian^1,4^, Xiaoyan Liu^3^, Yan Wang ^1^, Ying Liu^1^, Jie Ma^1^, Haidan Sun^3^, Jing Li^3^, Xiaoyue Tang^3^, Zhengguang Guo^3^, Wei Sun^3*^, Jishui Zhang^2*^, Wenqi Song^1,4*^

^1^ Department of Clinical Laboratory, Beijing Children's Hospital, Capital Medical University, National Center for Children's Health, Beijing, China, 100045

^2^ Department of Mental Health, Beijing Children's Hospital, Capital Medical University, National Center for Children's Health, Beijing, China, 100045

^3^ Institute of Basic Medical Sciences, Chinese Academy of Medical Sciences, School of Basic Medicine, Peking Union Medical College, Beijing, China, 100005

^4^ Beijing Advanced Innovation Center for Big Data-Based Precision Medicine, Beihang University & Capital Medical University, Beijing, China, 100083

***Corresponding author:**

Prof. Wei Sun, E-mail: sunwei@ibms.pumc.edu.cn; Tel.: 0086-010-69156995

Prof. Jishui Zhang, E-mail: zhangjishui@163.com; Tel.: 0086-010-59616927

Prof. Wenqi Song, E-mail: songwenqi1218@163.com; Tel.: 0086-010-59616962

**Supplementary Figure Caption**

**Fig. S1** Assessment of QC samples

Trend plot shows the variation of t [1] over all QC Samples. X axis numbers represents sample number, Y axis is arbitrary (3 SD); Green dots, QC samples

**Fig. S2** Analysis of metabolic profiling variation of gender in different age groups

Score plot of unsupervised PCA, OPLS-DA and validate model (model validation by resampling 1000 times under the null hypothesis) of urinary metabolic profiling between male and female children in five different age groups

**Fig. S3** Analysis of metabolic profiling variation of ages. Score plot of PCA and validation models of urinary metabolic profiling for male and female children with five different age groups

**Fig. S4** Analysis of metabolic profiling in ADHD disease group as compared with control group.

1. Score plot of PCA model based on metabolome between disease and control group.
2. Score plot of OPLS-DA and validation models based on metabolome between disease and control group.
3. ROC plot with 10-fold cross-validation based on model of FAPy-adenine, N-Acetylaspartylglutamic acid and Dopamine 4-sulfate to quantify the discrimination degree of disease and control group

**Fig. S5** Analysis of metabolic profiling in ADHD disease group and normal control. Score plot of OPLS-DA and validation model of urinary metabolic profiling.

1. ADHD without tic disorder and normal controls.
2. ADHD with tic disorder and normal controls.

**Fig. S6** ROC with 10-fold cross-validation

1. ROC of training set based on model of FAPy-adenine, 3-methylazelaic acid and phenylacetylglutamine to quantify the discrimination of ADHD disease and healthy control.
2. ROC of training set based on panel included FAPy-adenine, N-Acetylaspartylglutamic acid, dopamine 4-sulfate, aminocaproic acid and asparaginyl-leucine to discriminate ADHD comorbid tic disorders and healthy control

**Fig. S7** Metabolic characteristics for three groups including ADHD without tic disorder, ADHD comorbid tic and healthy control. Metabolic pathways were enriched based on metabolites with the highest level in each group. The KEGG database was the background pathway database
